# Angiopoietin-1, Angiopoietin-2 and Bicarbonate as Diagnostic Biomarkers in Children with Severe Sepsis

**DOI:** 10.1371/journal.pone.0108461

**Published:** 2014-09-25

**Authors:** Kun Wang, Vineet Bhandari, John S. Giuliano, Corey S. O′Hern, Mark D. Shattuck, Michael Kirby

**Affiliations:** 1 Department of Mathematics, Colorado State University, Fort Collins, Colorado, United States of America; 2 Department of Mechanical Engineering & Materials Science, Yale University, New Haven, Connecticut, United States of America; 3 Department of Pediatrics, Yale University School of Medicine, New Haven, Connecticut, United States of America; 4 Department of Applied Physics, Department of Physics, and Graduate Program in Computational Biology & Bioinformatics, Yale University, New Haven, Connecticut, United States of America; 5 Benjamin Levich Institute and Physics Department, The City College of New York, New York, New York, United States of America; D′or Institute of Research and Education, Brazil

## Abstract

Severe pediatric sepsis continues to be associated with high mortality rates in children. Thus, an important area of biomedical research is to identify biomarkers that can classify sepsis severity and outcomes. The complex and heterogeneous nature of sepsis makes the prospect of the classification of sepsis severity using a single biomarker less likely. Instead, we employ machine learning techniques to validate the use of a multiple biomarkers scoring system to determine the severity of sepsis in critically ill children. The study was based on clinical data and plasma samples provided by a tertiary care center's Pediatric Intensive Care Unit (PICU) from a group of 45 patients with varying sepsis severity at the time of admission. Canonical Correlation Analysis with the Forward Selection and Random Forests methods identified a particular set of biomarkers that included Angiopoietin-1 (Ang-1), Angiopoietin-2 (Ang-2), and Bicarbonate (HCO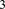) as having the strongest correlations with sepsis severity. The robustness and effectiveness of these biomarkers for classifying sepsis severity were validated by constructing a linear Support Vector Machine diagnostic classifier. We also show that the concentrations of Ang-1, Ang-2, and HCO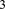 enable predictions of the time dependence of sepsis severity in children.

## Introduction

Pediatric sepsis continues to be a very significant cause of mortality in children [1], [2]. Patients who develop organ dysfunction (i.e. severe sepsis or septic shock) have worse morbidity and mortality compared to those who do not [3], [4]. Diagnosing and classifying the severity of sepsis is a significant challenge due to the highly variable and nonspecific nature of the signs and symptoms of sepsis. Biomarkers that play critical roles in the disease process show great promise in indicating the severity of sepsis. There are many biomarkers that have been studied for potential use in the early diagnosis and classification of sepsis [5], [6]. However the complex and heterogeneous nature of sepsis makes the prospect of single biomarker classification less likely.

No single biomarker has sufficient specificity or sensitivity to be routinely employed in clinical practice. A combination of several sepsis biomarkers may be more effective, as has been suggested by other investigators [7]–[9]. Multivariate methods have the advantage of selecting an optimal subset of variables from a large number of variables and taking into account the relationship among the selected variables based on a specific outcome.

In this manuscript, we employ a discovery-oriented approach to identify a panel of diagnostic biomarkers. We systematically evaluate many commonly obtained clinical parameters and laboratory values using the multivariate diagnostic capacity of a scoring system that incorporates 17 potential variables to classify patients admitted to a tertiary care center's Pediatric Intensive Care Unit (PICU) with or without sepsis (PICU/sepsis group) versus those with severe sepsis (PICU severe sepsis group).

## Materials

### Study population

This study was approved by the Pediatric Protocol Review Committee and the Human Investigation Committee at Yale University School of Medicine. Patient records were anonymized and de-identified prior to analysis. The biological specimens and clinical data sets were obtained from a prospective observational study of critically ill pediatric patients with varying degrees of sepsis severity conducted at a tertiary care center PICU during the time period 9/2009–12/2011 [10].

All patients admitted to the PICU were evaluated for eligibility. Forty-five patients met the eligibility criteria and consented to participate in the study. Using the 2005 pediatric sepsis and organ dysfunction definitions [11], patients were divided into one of five categories based on clinical exam findings in the first 24-hours of PICU admission. The categories included systemic inflammatory response syndrome (SIRS), non-SIRS, sepsis, severe sepsis and septic shock. Briefly, SIRS required the presence of at least two of the following four criteria with one being abnormal temperature or leukocyte cout: abnormal core temperature, mean respiratory rate, leukocyte count, or tachycardia. Non-SIRS patients were admitted to the PICU but did not meet SIRS criteria. Patients with sepsis fulfilled SIRS criteria with suspected or proven infection. Patients with severe sepsis met the criteria for sepsis with organ failure, and septic shock patients were a subset of the severe sepsis group with cardiovascular organ failure [11]. Blood samples were collected every 12 hours for the first 3 days and then once a day for the last 4 days. Data collection was discontinued when the patient was discharged from the PICU. A maximum of 10 samples for 7 days were obtained from each patient. As a result of PICU discharge and line removal, the total number of samples available for analysis decreased with time for all patient groups. The number of samples for each time point is shown in Figure S1. Commercial enzyme-linked immunosorbent assay (ELISA) kits were used to measure plasma levels of Ang-1 and Ang-2. Descriptive data consisting of demographics and clinical data for all patients included in the clinical studies are provided in Tables S1 and S2 in File S1. Additional details can be found in Text S1 in File S1 and Ref. [10].

### Biomarkers

To create a robust model of a specific combination of biomarkers for predicting the severity of sepsis in children in an unbiased manner, we selected multiple clinical and laboratory variables from the database of our study [10]. These 17 variables are as follows: (1) Age, (2) Weight (Wgt), (3) admission Pediatric Index of Mortality 2 (PIM-2) [12], (4) White Blood Cell count (WBC), (5) Hemoglobin count (Hgb), (6) Hematocrit (Hct), (7) Platelet count (Plt), and the levels of (8) Sodium (Na), (9) Potassium (K), (10) Chloride (Cl), (11) HCO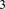, (12) Blood Urea Nitrogen (BUN), (13) Creatinine (Cr), (14) Ang-1, (15) Ang-2, (16) Ang-2/Ang-1 ratio, and (17) Vascular Endothelial Growth Factor (VEGF). To validate the data analysis, we augmented this data set to include (18) Gaussian distributed noise (g-Noise) and (19) uniformly distributed noise (u-Noise). These 19 variables were then used to develop sepsis severity prediction models.

### Statistical analysis

Patients were classified within the first 24 hours of PICU admission into the five categories listed above based on the 2005 pediatric sepsis and organ dysfunction definitions [11]. We further consolidated these into the following two categories: 1) the PICU/sepsis group (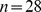) included those not meeting SIRS criteria but were admitted to the PICU (non-SIRS) (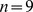), SIRS (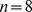), and sepsis (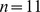); and 2) the PICU severe sepsis group (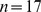) included those with severe sepsis (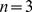), and septic shock (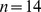). For the original study listed in Ref. [10], a two-sided Mann-Whitney test estimated a sample size of 50 (10 patients per group) to detect 1.5–1.8 standard deviations in the level of Ang-2 between comparison groups, assuming a standard deviation of 1,500 pg/mL, power of 80%, and a significance level (alpha) of 0.05.

## Methods

### Data Preprocessing

Our dataset (input), a 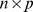 real-valued matrix 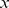, contains 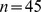 attributes and 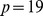 biomarkers. Since the range of values of the biomarkers varies widely, it should be normalized so that each biomarker contributes approximately proportionately. We normalized 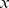 to have zero mean and unit standard deviation for each biomarker [13]: 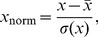
(1)where 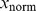 is a 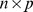 matrix, 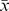 and 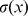 are the mean value and standard deviation of 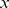 for each biomarker. We also assigned each attribute 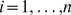, a sepsis severity score, 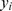. 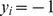 is given to each in the PICU/sepsis group and 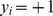 for the PICU severe sepsis group.

### Canonical correlation analysis

CCA finds linear combinations of variables between two sets of data, 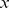 and 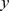 in our study, which have maximum correlation with each other [14], [15]. Here we selected the optimal subset of biomarkers 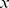 that has the maximum correlation with 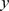 for 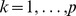, by calculating the correlations between all possible 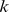-combinations of 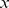 and 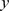. The results are displayed in Table 1.

**Table 1:** Stepwise Biomarker Selection using Canonical Correlation Analysis, Forward Selection and Random Forests.

Dim	Corr	Entering	Leave	Forward Selection	Random Forests
1	0.3811	Ang-2		Ang-2	Ang-2/Ang-1
2	0.4772	Ang-1		Ang-1	HCO3
3	0.5501	HCO3		HCO3	Ang-2
4	0.5842	Plt		Plt	Ang-1
5	0.6079	Age		Age	Cl
6	0.6183	Cl		WBC	PIM-2
7	0.6221	BUN, Hct, WBC	Cl, HCO3	Hct	Age
8	0.6286	VEGF		BUN	K
9	0.6311	PIM-2		VEGF	Hgb
10	0.6359	Cl, HCO3	PIM-2	PIM-2	VEGF
11	0.6395	Cr, Wgt	Age	g-Noise	Wgt
12	0.6409	Hgb, Na, Age	Cl, Wgt	Cl	Na
13	0.6414	Ang-2/Ang-1		Cr	g-Noise
14	0.6419	Wgt		u-Noise	Plt
15	0.6424	PIM-2		Ang-2/Ang-1	WBC
16	0.6427	Cl, u-Noise	Na	Hgb	u-Noise
17	0.6429	K		Wgt	Cr
18	0.6429	Na, g-Noise	K	K	BUN
19	0.6430	K		Na	Hct

We apply Canonical Correlation Analysis for all possible 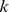-combinations (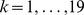) to determine the subset of 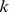 biomarkers with the highest correlation with the sepsis severity score. The ‘Enter’ column indicates the biomarker that is added to achieve the highest correlation at each 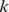. The ‘Leave’ column indicates the biomarker that is eliminated from the combination at each 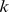. A biomarker will stay in the combination until it occurs in ‘Leave’ column. The ‘Forward Selection’ column gives the biomarker selected by the Forward Selection method when applied one biomarker at a time. The ‘Random Forests’ column gives the biomarker ranked by the mean decrease in accuracy measured by the Random Forests method.

### Linear support vector machines

In machine learning, a linear support vector machine (SVM) is a learning model used for classfication and regression analysis [16]. A SVM model separates two categories by a hyper-plane that has maximum margin for a given training dataset. New attributes are predicted to belong to a category based on which side of the hyper-plane they fall on.

The hyper-plane can be described by the equation: 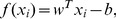
(2)where 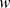 is the normal vector to the hyper-plane, 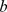 is the offset of the hyper-plane from the origin, and 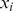 is a 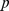-dimensional vector of normalized biomarker values for attribute 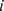 in our study. The search of this hyper-plane can be translated into the following optimization problem: 
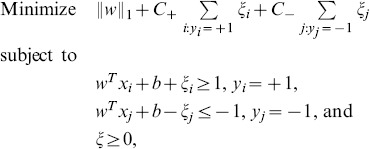
(3)where 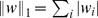 is the 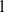-norm of a vector, which induces the sparsity in the weight vector 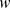 [17]. The slack variable, 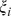, measures the degree of misclassification of 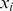. The parameters 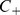 and 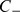, which determine the penalty assigned to the total error from misclassified samples, are chosen so that 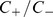 is given by the ratio of the number of negative and positive training evaluations with 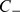 = 1.0.

### Ensemble method

Due to the limited size and noise of our data, we follow the training procedure in Ref. [18]. A random one-third of the data is selected as test set, 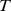. The remaining data is used as training set, 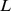. Bagging is used to construct the classifiers ensemble. Each new training set, 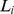, is drawn, with replacement, from the original training set, 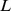. Then a classifier, SVM or tree, is constructed on this new training set, 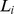. In this study, we construct a classifiers ensemble 50 times, 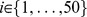. The final classification is obtained by calculating the mean of the ensemble of 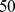 classifiers. This procedure is repeated 100 times and statistical measures on 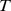 are averaged.

### Calculation of statistical measures

TPR, TNR, NPV, and PPV are statistical measures of the predictive performance of a binary classification test. TPR (or sensitivity) measures the proportion of actual positives that are correctly identified. TNR (or specificity) measures the proportion of actual negatives that are correctly identified. PPV (or precision) measures the proportion of positives that are true positive. NPV measures the proportion of negatives that are true negatives.

These statistical measures are calculated for each one of the 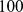 random divisions of test sets 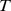 by the classifier built on the bootstrap aggregation method. Their mean and standard error are calculated from the groups obtained from the 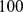 random divisions.

## Results

### Biomarkers selection

Feature selection is an important part of the data analysis given the fact that the data contains many redundant or irrelevant features. Redundant features provide no additional information than the selected features, and irrelevant features provide no useful information. Feature selection is widely used in data sets with abundant features but comparatively few samples. In machine learning and statistics, the goal of a feature selection method is to select an optimal subset of relevant features for model construction.

In this study, there are 17 variables (features) augmented by 2 variables consisting of Gaussian and uniform noise to provide a baseline check for the data analysis. From the univariate correlation analysis, we found that this data set contained several possible redundant biomarkers and, not surprisingly, at least two irrelevant features (g-Noise and u-Noise). To extract an optimal subset of biomarkers, we analyzed the multivariate correlation between the outcome, sepsis severity score (0 for PICU/sepsis and 1 for PICU severe sepsis), and the input, which is a subset of variables.

**Figure 1:**
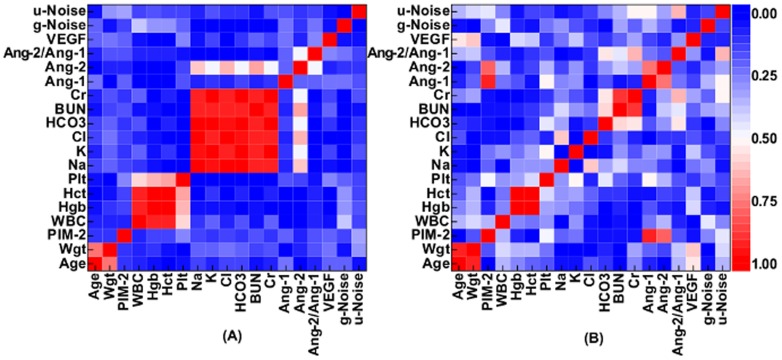
Heatmaps of pairwise correlations. Heatmaps of all pairwise correlations between the 17 variables (plus two noise samples) for patients in the (A) PICU/sepsis and (B) PICU severe sepsis groups. The color scale from blue to red indicates increasing correlations between the pair of biomarkers at the corresponding locations on the horizontal and vertical axes.

A comparison of the univariate correlations for these two groups is shown in Fig. 1. The univariate analysis revealed that Na, K, Cl, HCO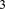 form a group of highly correlated biomarkers (with correlations that range from 0.937 to 0.998) for the PICU/sepsis group. However, these variables are not strongly correlated for the PICU severe sepsis group (with correlations that range from 0.001 to 0.608). This notable difference between the PICU/sepsis and PICU severe sepsis groups indicates that these biomarkers may not independently provide information about the sepsis severity diagnosis. We also note that Ang-1 and Ang-2 are highly correlated with each other in the PICU severe sepsis group (0.76), but this correlation is significantly reduced for the PICU/sepsis group (0.21). Meanwhile, Ang-2/Ang-1 does not correlate very strongly with either Ang-1 (0.21 in PICU/sepsis, 0.24 in PICU severe sepsis) or Ang-2 (0.48 in PICU/sepsis, 0.17 in PICU severe sepsis). Based on these observations, we seek to identify an optimal set of non-redundant variables and biomarkers to predict the severity of sepsis.

In our recent study [19], we found that canonical correlation analysis (CCA) [14], [15], [20] can be applied effectively to identify an optimal subset of biomarkers with the maximum correlation with the outcome. As shown in Table 1, we found that the subset of Ang-2, Ang-1, and HCO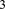 maximizes the correlation with the sepsis severity score. As expected, the two forms of random noise are selected near the end of the process when the correlation saturates for large subsets. We also applied the forward selection (FS) method to identify the optimal subset of biomarkers. FS is a greedy algorithm that adds the best feature at each step [21], [22]. We found that the performance of the subset of biomarkers selected by FS was similar to that selected by CCA on this data set.

### The optimal subset

In this study, we built a diagnostic classifier by selecting the subset of 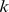 biomarkers with the best diagnostic performance for each value of 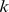. For each 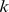, we applied the ensemble method [18], [23] to construct a linear support vector machine (SVM) classifier [24] for the CCA-selected subset of biomarkers. SVM [17] finds a decision function that separates the high-dimensional data with the maximum margin. To quantify the classifier performance, we calculated the true positive rate (TPR), true negative rate (TNR), positive predictive value (PPV), and negative predictive value (NPV). See the Methods section for details.

**Figure 2:**
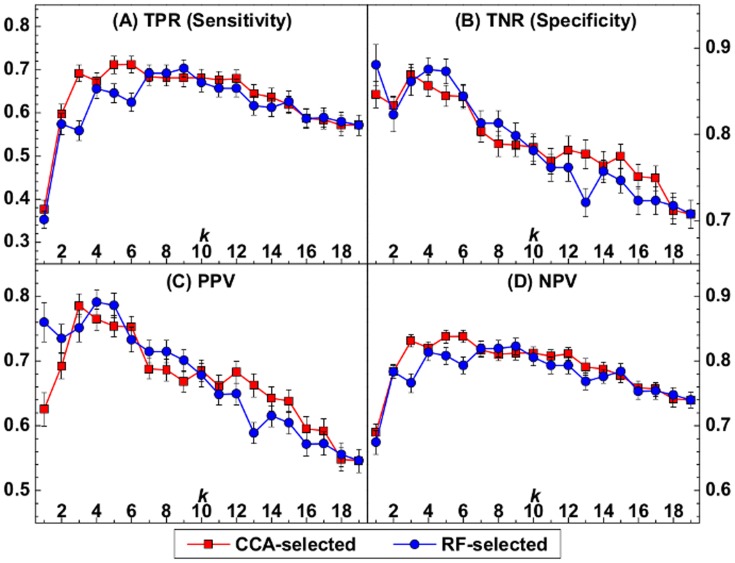
Prediction measures obtained from the Support Vector Machine (SVM) using the 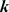-combinations selected by the Canonical Correlation Analysis (CCA) and Random Forests (RF) methods. The prediction measures (A) true positive rate (TPR), (B) true negative rate (TNR), (C) positive predictive value (PPV), and (D) negative predictive value (NPV) are shown for each step 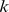. For each 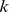, a SVM ensemble with bagging is constructed based on the CCA- and RF-selected subset of biomarkers.

In Figure 2, we find that all statistical measures reach a peak or saturate near 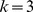 using the CCA-selected biomarkers, Ang-2, Ang-1, and HCO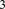, which suggests that these three biomarkers are the optimal subset for our data set (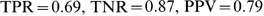, and 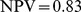 at 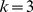). By adding HCO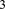 to the optimal subset from 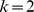 to 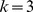, the combination has higher TPR (0.60 at 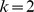 versus 0.69 at 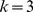) and PPV (0.69 at 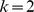 versus 0.79 at 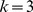) when compared to the combination of Ang-2 and Ang-1. TNR (0.84 at 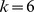 versus 0.80 at 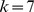) and PPV (0.75 at 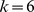 versus 0.69 at 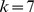) begin to decrease from their plateau values when HCO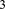 leaves the subset at 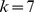. The improvement at 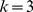 and decrease at 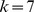 indicate the diagnostic importance of HCO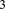.

### Redundant biomarkers

Recent studies [10], [25]–[27] suggest that plasma levels of Ang-2 and Ang-1 can serve as clinically informative biomarkers of sepsis severity. Further, the Ang-2/Ang1 ratio is considered to be a more relevant sepsis severity biomarker than isolated levels of each biomarker because of their antagonistic roles in regulating the tyrosine kinase receptor, Tie-2 [27]. However, both of our biomarker selection methods, CCA and FS, select Ang-2/Ang-1 to the optimal subset relatively late, *i.e.*, at large 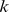 (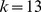 and 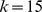) as shown in Table 1. This suggests that a combination of Ang-2, Ang-1, and HCO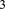, is potentially more effective than using the ratio of Ang-1 and Ang-2 with other biomarkers.

It is also interesting to consider the univariate and bivariate performance of these biomarkers. This analysis provides additional insight into the relative performance of different subsets of biomarkers and how they work together to provide inferences.

**Figure 3:**
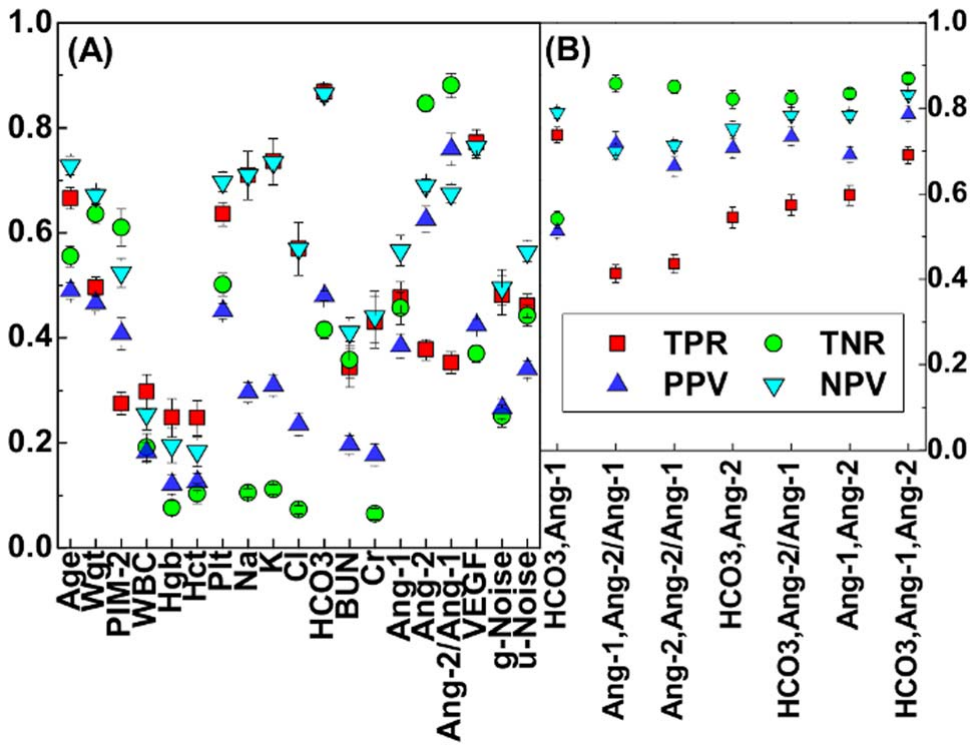
Prediction measures for single and pairs of biomarkers from the Support Vector Machine (SVM). True positive rate (TPR), true negative rate (TNR), positive predictive value (PPV), and negative predictive value (NPV) are shown for (A) each single biomarker and (B) all pairwise combinations of Ang-1, Ang-2, HCO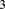 and Ang-2/Ang-1. The prediction measures for the CCA-selected optimal subset of biomarkers at 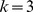 (Ang-2, Ang-1, and HCO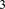) are also shown in (B) for comparison.

In Fig. 3(A), the relative performance of the univariate biomarkers performance is shown: 1) Ang-1 has consistent performance for all statistical measures compared to other biomarkers (see Table 2), 2) Ang-2 has a high TNR (0.85) and PPV (0.63) but relatively low TPR (0.38), and 3) HCO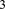 has the highest TPR (0.87) and NPV (0.86) but relatively low TNR (0.42) and PPV (0.48). These observations indicate that the performances of these biomarkers did not correlate with each other. This supports the observation that the best subset of biomarkers includes both Ang-1 and Ang-2 since they provide distinct information. We also show that the combination of Ang-2, Ang-1 and HCO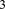 improves the predictive capability by reducing overfitting in Fig. 2. The performance for the CCA-selected subsets decreases when 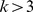.

**Table 2:** Prediction measures for single biomarker from Support Vector Machine.

Variable	TPR	TNR	PPV	NPV
Age	0.666	0.555	0.490	0.728
Wgt	0.496	0.636	0.466	0.671
PIM-2	0.276	0.611	0.407	0.524
WBC	0.298	0.192	0.183	0.255
Hgb	0.249	0.076	0.121	0.195
Hct	0.248	0.104	0.126	0.184
Plt	0.636	0.501	0.451	0.697
Na	0.710	0.105	0.297	0.710
K	0.737	0.112	0.309	0.735
Cl	0.570	0.073	0.236	0.570
HCO 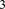	0.868	0.415	0.480	0.865
BUN	0.343	0.358	0.197	0.411
Cr	0.430	0.065	0.177	0.440
Ang-1	0.477	0.457	0.384	0.566
Ang-2	0.378	0.846	0.625	0.690
Ang-2/Ang-1	0.353	0.881	0.760	0.675
VEGF	0.773	0.370	0.424	0.764
g-Noise	0.481	0.251	0.266	0.496
u-Noise	0.461	0.442	0.340	0.564

True positive rate (TPR), true negative rate (TNR), positive predictive value (PPV), and negative predictive value (NPV) are shown for each single variable.

These results suggest, when examining groups of three, Ang-2/Ang-1 may be a redundant biomarker, *i.e.*, no additional information is gained when Ang-1 and Ang-2 data is known. We explore here how this ratio performs in isolation, *i.e.*, as a derived univariate statistic. We applied the same procedure as above to construct a SVM classifier for each single biomarker and show the statistical measures in Fig. 3(A). Overall, we find that Ang-2 and Ang-2/Ang-1 have comparable prediction performance (Fig. 3(A)). However, Ang-2/Ang-1 outperforms Ang-2 for PPV (0.76 for Ang-2/Ang-1, 0.63 for Ang-2), which suggests that Ang-2/Ang-1 alone may be a predictive biomarker. The similar performance of Ang-2 and Ang-2/Ang-1 suggest that these two biomarkers capture very similar information.

Of course it is not necessarily a fair assessment to compare true univariate biomarkers such as Ang-1 and Ang-2 to their ratio since this contains information from two measurements. Thus, we also compared the performance of combinations of Ang-1, Ang-2, HCO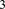, and Ang-2/Ang-1 in Fig. 3(B). The combination of Ang-2 and Ang-2/Ang-1 does not notably improve each predictive measure compared to these biomarkers alone, which also indicates that these two biomarkers are redundant. In contrast, the combination of Ang-1 and Ang-2 has notably higher NPV (0.78) and TPR (0.60) and comparable values for the other prediction measures compared to each single biomarker (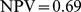 and 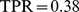 for Ang-2, 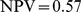 and 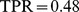 for Ang-1) and Ang-2/Ang-1 (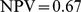 and 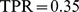). This suggests that the ratio Ang-2/Ang-1 is less effective than using Ang-1 and Ang-2 separately.

For completeness, we also show the performance for the CCA-selected optimal subset of three biomarkers HCO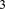, Ang-1 and Ang-2 on the far right of Fig. 3(B). This optimal subset notably improves the predictive capability as indicated by the small spread of values in the predictive measures.

### The diagnostic classifier

We applied the linear SVM ensemble method [23], [24] to construct a decision function using the CCA-selected optimal subset of biomarkers at 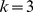: Ang-2, Ang-1, and HCO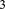. The optimal decision function is 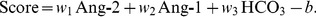
(4)


Table 3 provides the weights 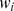, errors 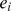, means 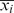 and standard deviations 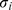 of the biomarkers. Since the range of values of the biomarkers varies widely, all values of the biomarkers are normalized by subtracting the mean and then dividing by the standard deviation in Eq. 4. See the Methods section for details. With this decision function, if the sepsis severity score (Score) is greater than or equal to zero, the severity diagnosis is 1, otherwise it is 0. The magnitudes of weights 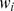 indicate the importance of the corresponding biomarker [28]. We find that Ang-2 has a larger weight than Ang-1 and HCO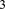, which is consistent with the results for the single biomarker classification in Fig. 3(A), where the TNR, and PPV are larger for Ang-2 than Ang-1 and HCO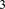. However, the TPR and NPV are larger for HCO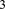 compared to that for Ang-2. The sign of each weight 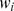 indicates the sign of the correlation of the biomarker with the sepsis severity score. Thus, the sepsis severity score for a patient with a relatively high Ang-2 level and low Ang-1 and HCO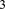 levels is most likely positive. This relation between biomarkers and sepsis severity score has been observed in clinical studies [25], [29], [30].

**Table 3:** Parameters for the decision function that includes the CCA-selected optimal subset of biomarkers at 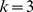.

i	Biomarker	Mean	Standard Deviation	Weight	Standard Error of Weight
		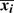	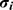	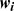 ( 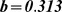 )	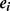
1	Ang-2	8518.1	13264	1.994	0.065
2	Ang-1	2649.2	4008.9	−1.396	0.050
3	HCO 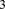	27.270	24.361	−1.340	0.072

The values of the weights 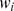, errors 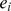, means 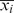, and standard deviations 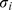 for the biomarkers in Eq. (4).

### Longitudinal measurements of the predictor

A linear SVM finds the hyper-plane that separates data with maximum margin by categories. In our study, the sign of the sepsis severity score (Score) in Eq. 4 can predict the category for a patient. The magnitude of the Score represents the distance from the decision boundary and indicates the severity of sepsis. A large positive Score indicates critical severity.

**Figure 4:**
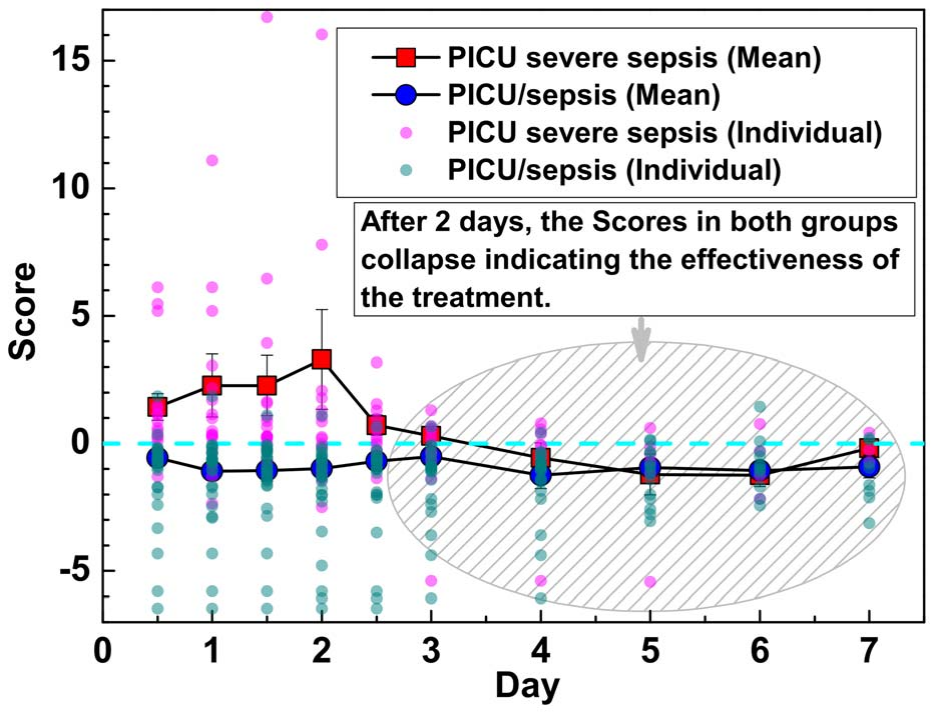
Longitudinal measurements of the sepsis severity score. The sepsis severity scores (Score) for patients from the PICU/sepsis group and the PICU severe sepsis during the 7 days of illness. Both the mean and individual severity scores are plotted.

Based on the fact that patients were hospitalized during the study, the longitudinal measurements should show a decrease in the number of patients in the PICU severe sepsis group. Fig. 4 shows that Scores in the PICU severe sepsis group are notably separated from the PICU/sepsis group for the first two days after admission. After two days, the Scores in the PICU severe sepsis group decrease and collapse with those from the PICU/sepsis group indicating the effectiveness of the treatment. Additionally, the sepsis severity score (Eq. 4) measured on the first 2 days after admission may allow for the early identification of patients with severe sepsis, which is important for the initiation of early goal-directed therapies.

### Comparison with the random forests learning method

Random forests (RF) [31] is an ensemble method [18], [23], which grows multiple classification and regression trees (CART) [32] for prediction. Every tree in the forests is constructed by a random selected bootstrap training set with replacement [18]. The splitting criteria for every decision node in a tree are also chosen from a random subset of the features without replacement. With the replacement from the original data, about two-thirds of the samples are used to construct a tree [18]. The out-of-bag (OOB) data, which are not chosen in the construction, are then used to estimate the prediction accuracy and the importance of the features [31], [33]. Unlike a linear SVM, which constructs a hyper-plane to classify the data, a tree is a hierarchical classification procedure, which recursively partitions the data to increase the purity of the nodes with respect to the outcome [32].

RF provides two measures, the mean decrease in accuracy (MDA) and mean decrease in the Gini index [31], [33], to estimate the importance of the features. In our study, the MDA is chosen to estimate the feature importance since the decrease in the Gini index is not as reliable as MDA [33], [34]. By randomly permuting the values of a given feature in the OOB data for each tree, RF measures the accuracy difference between untouched and permuted OOB data. The average of this accuracy difference over all trees in the forest is the MDA for the given feature. The MDA is the average increase in misclassification rate due to the permutations. The larger the MDA the more important the corresponding feature is with respect to the outcome.

**Figure 5:**
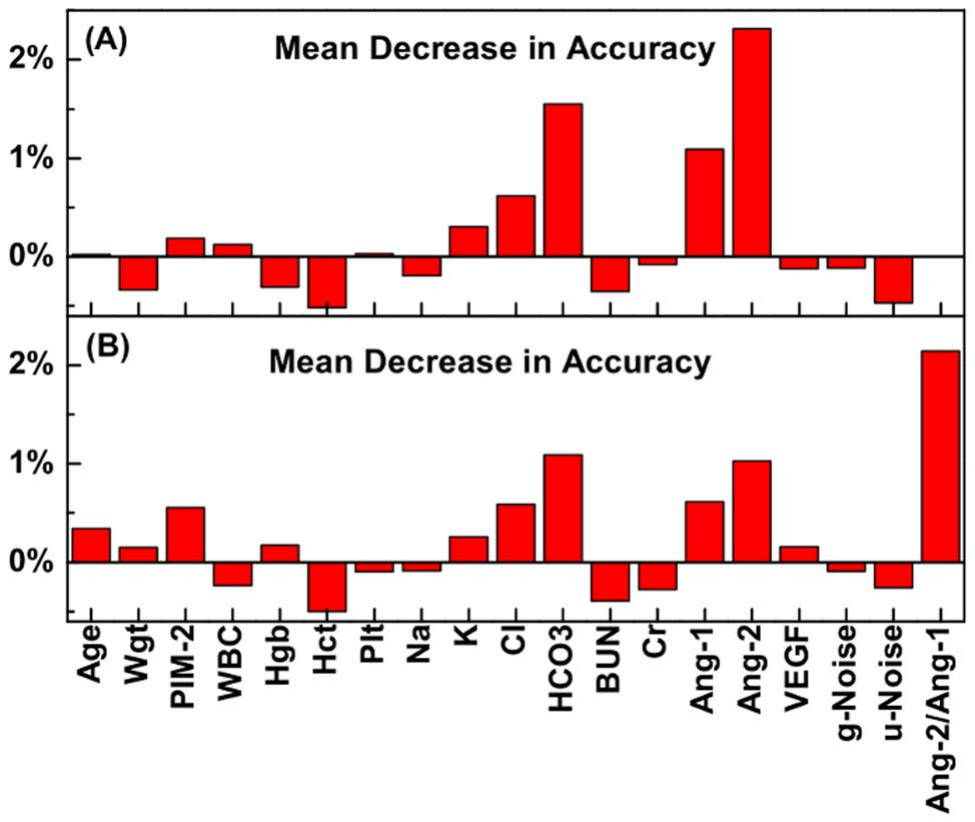
Measures of the biomarker importance obtained from the Random Forests method. Mean Decrease in Accuracy (MDA) are shown for biomarkers in (A) without Ang-2/Ang-1 and (B) with Ang-2/Ang-1 using the Random Forests method with 1,000 trees for each.

Following Ref. [31], we construct a forest with 1,000 trees to estimate the MDA for the biomarkers. We generated two RF: one for which Ang-2/Ang-1 is excluded (Fig. 5(A)) or included (Fig. 5(B)). Because of the interaction of Ang-2, Ang-1, and Ang-2/Ang-1, the existence of Ang-2/Ang-1 suppresses the importance of Ang-2 and Ang-1. However, both CCA and FS methods tend to select the combination of Ang-2 and Ang-1 as the most predictive feature. We notice that HCO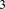 is considered important for all three methods, which suggests HCO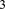 is also an important biomarker. The RF ranked biomarkers based on the importance are also shown in Table 1.

We also constructed a SVM ensemble using the RF-selected subset for each step 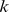 in Fig. 2 for comparison. Similar to the CCA-selected subset in Fig. 2, all prediction measures saturate at 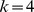 and decrease for 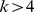. We find that the RF-selected optimal subset, Ang-2/Ang-1, HCO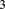, Ang-2, Ang-1 at 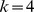, have comparable prediction performance with the CCA-selected optimal subset at 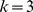.

## Discussion

In this study, we employed machine learning approaches to analyze the clinical data of children with severe sepsis using feature selection methods, such as CCA, SVM, FS and RF. Feature selection methods are helpful in identifying biomarkers with minimum redundancy that can be useful in clinical diagnosis. Our multivariate feature selection methods select the combination of Ang-1, Ang-2, and HCO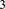 as the optimal biomarkers for our data set. We demonstrated that this optimal combination of biomarkers significantly outperformed each single biomarker and all other combinations with redundant or irrelevant biomarkers for all statistical measures.

Our work [10], [27], and that of others, has shown the biological plausibility and clinical relevance of Ang-2 and Ang-1 levels in PICU patients with severe sepsis. It is interesting to note that combining Ang-2 and Ang-1 with a well-established (and routinely measured) indicator of an imbalance in the acid-base levels performs much better than other scoring systems that are more complex (for example, PIM-2 [12]).

Our data driven approach indicates that there is an optimal set of biomarkers for diagnosing severe sepsis. We have demonstrated that the use of additional biomarkers actually reduces the quality of the diagnostic scoring system. This is a potentially important observation in the sense that it suggests that more feature rich data may not be helpful, but actually harmful to patient care.

In addition, a sepsis severity score function (Eq. 4) using this optimal combination of biomarkers was constructed by the SVM ensemble method. With this function, we can interpret the relation between these three biomarkers and the sepsis severity from the associate weights, 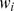 [28]. Even though these relations have been observed in clinical studies [10], [25], [26], we assert that our methodology is useful since it obtains similar results to those of the clinical studies using unbiased, rigorous statistical analyses. It also holds promise for the discovery of novel biomarkers.

The proposed sepsis severity score for each sample is also evaluated during the treatment. The patients in the PICU severe sepsis group have significantly high severity scores after admission. The sepsis severity scores measured on the first 2 days after admission may allow for the early identification of patients with severe sepsis. After two days treatment, the severity scores for each patient decline and collapse to match patients without severe sepsis. Based on the fact that all patients survived hospitalization, the change in the longitudinal measurements of this score function validates the robustness and effectiveness of this function as regards its potential utility at different stages of treatment.

It has been observed that single biomarkers, in isolation, have limited diagnostic capacity [5]. This study supports this conclusion. Our analysis strongly supports the conclusion that a combination of different biomarkers is more effective, i.e., using multiple biomarkers for diagnosis is superior to drawing conclusions from single biomarkers. The rationale for this observation may be that the biomarkers are not independent of each other but, as we have shown with our canonical correlation analysis, are correlated in groups. The identification of an optimal combination of biomarkers allows clinicians to focus on a small subset of indicators, which simplifies the diagnosis of sepsis in children with a spectrum of severities.

Despite the success in the classification of sepsis severity for this patient group, our study has several limitations. First, the data set was obtained from a single institution making generalizability difficult. Second, the biomarkers used to construct our models were based on clinical availability for most patients. It is possible that additional biomarkers, such as cytokines, would have improved the statistical measures for our models. Finally, since we have shown that measures of acid-base status are predictive biomarkers, it is likely that other acid-base determinants from blood gas analyses will also be predictive biomarkers. However, blood gas results were only available for the severe sepsis group (not other groups), and thus including blood gas measurements would have biased our findings. We advocate new clinical studies that include additional clinical variables, such as blood gas panels, to address the question of finding the most predictive set of biomarkers for severe sepsis.

In conclusion, we have shown that a linear additive combination of 3 biomarkers, namely Ang-2, Ang-1 and HCO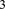 provides a robust prediction of sepsis severity in patients admitted to the PICU. Additional independent studies are needed to confirm or refute the clinical utility of our biomarker combination for sepsis severity prediction. The collection of data sets with larger sample sizes would also be very useful for validating our statistical study.

## Supporting Information

Figure S1 **Sample size by study day.** Samples were obtained twice per day for the first 3 days and then once per day for the last 4 days, for a maximum of 7 days and 10 samples. Sample collection was discontinued when the patient was discharged from the PICU, after the 7-day study completion, or when the clinical team deemed it unnecessary to draw further labs for patient care.
(TIF)

File S1 Contains Table S1, Infectious organisms: Causative organisms isolated in patients. N gives the number of patients with a given proven infection. Table S2, Baseline patient characteristics: Statistical analysis of the baseline patient characteristics based on the evaluation distributions of the PICU/sepsis group and PICU severe sepsis group. Categorical variables, presented as count (percentage), were analyzed using Fisher exact test. Continuous variables, presented as mean (standard deviation), were analyzed using the two-tailed t test. P values are comparisons between two groups. Any significance level of P less than 0.05 is associated with the diagnosis. Text S1, Supplementary Text.
(PDF)
