# Supplementary figures and images for: Angiopoietin-1, Angiopoietin-2 and Bicarbonate as Diagnostic Biomarkers in Children with Severe Sepsis
Source: PLoS One. 2014 Sep 25;9(9):e108461. doi: 10.1371/journal.pone.0108461 (PMC4178003; doi:10.1371/journal.pone.0108461)

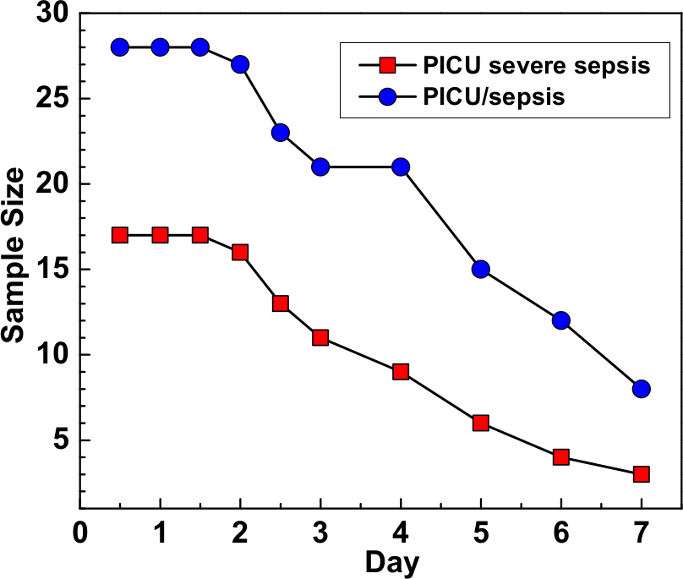

Supplement: Figure S1 — Sample size by study day. Samples were obtained twice per day for the first 3 days and then once per day for the last 4 days, for a maximum of 7 days and 10 samples. Sample collection was discontinued when the patient was discharged from the PICU, after the 7-day study completion, or when the clinical team deemed it unnecessary to draw further labs for patient care. (TIF) [file pone.0108461.s001.tif]
