# Supplementary material for: Angiopoietin-1, Angiopoietin-2 and Bicarbonate as Diagnostic Biomarkers in Children with Severe Sepsis
Source: PLoS One. 2014 Sep 25;9(9):e108461. doi: 10.1371/journal.pone.0108461 (PMC4178003; doi:10.1371/journal.pone.0108461)
Supplement: File S1 — Contains Table S1, Infectious organisms: Causative organisms isolated in patients. N gives the number of patients with a given proven infection. Table S2, Baseline patient characteristics: Statistical analysis of the baseline patient characteristics based on the evaluation distributions of the PICU/sepsis group and PICU severe sepsis group. Categorical variables, presented as count (percentage), were analyzed using Fisher exact test. Continuous variables, presented as mean (standard deviation), were analyzed using the two-tailed t test. P values are comparisons between two groups. Any significance level of P less than 0.05 is associated with the diagnosis. Text S1, Supplementary Text. (PDF) [file pone.0108461.s002.pdf]

Angiopoietin-1, Angiopoietin-2 and Bicarbonate as Diagnostic Biomarkers in Children with Severe Sepsis Supporting Information

Kun Wang[1,2], Vineet Bhandari[3], John S. Giuliano Jr[3], Corey S. O'Hern[2,4], Mark D. Shattuck[5], Michael Kirby[1,*]

1 Department of Mathematics, Colorado State University, Fort Collins, CO, USA

2 Department of Mechanical Engineering & Materials Science, Yale University, New Haven, CT, USA

3 Division of Perinatal Medicine, Department of Pediatrics, Yale University School of Medicine, New Haven, CT, USA

4 Department of Applied Physics, Department of Physics, and Graduate Program in Computational Biology & Bioinformatics, Yale University, New Haven, CT, USA

5 Benjamin Levich Institute and Physics Department, The City College of New York, New York, NY, USA

∗ **E-mail: kirby@math.colostate.edu**

⋄ **Referred to as Text S1 in the main text.**

Supplementary Text

Patient Recruitment and Classification

We performed a prospective observational study of critically ill pediatric patients with varying degrees of sepsis severity. Informed consent was obtained from parents,and assent was obtained from subjects when appropriate. Due to the maximum volume of blood potentially collected, patients weighing less than 10 kg or with hematocrits levels less than 25% were excluded. Since we were attempting to measure Angiopoietin levels over time, patients without anticipated blood draws or with an anticipated PICU length of stay less than 48 hours were also excluded [1]. Additionally, patients receiving or having received steroids within the previous 7 days were excluded due to their potential effect on biomarker levels.

Patients were classified into the PICU/sepsis group and the PICU severe sepsis group based on the 2005 pediatric sepsis and organ dysfunction definitions [2] as:

1. SIRS: Patients that meet at least two of the four criteria given in Table 2 of Ref. [2]

2. Sepsis: SIRS in the presence of or as a result of suspected or proven infection

3. Severe Sepsis: Sepsis plus one of the following: Cardiovascular organ dysfunction, acute respiratory distress syndrome, or two or more other organ dysfunctions.

4. Septic Shock: Sepsis and cardiovascular organ dysfunction as defined in Table 4 of Ref. [2]

References

1. Aghai Z, Faqiri S, Saslow J, Nakhla T, Farhath S, et al. (2007) Angiopoietin 2 concentrations in infants developing bronchopulmonary dysplasia: attenuation by dexamethasone. Journal of Perinatology 28: 149–155.

2. Goldstein B, Giroir B, Randolph A, et al. (2005) International pediatric sepsis consensus conference: Definitions for sepsis and organ dysfunction in pediatrics. Pediatric critical care medicine 6: 2–8.

Supplementary Figures and Tables

This section contains the supplementary figures and tables referred to in the main text.

Table 1. Infectious organisms

Infectious Class	Organism
Gram positive bacteria (N)	Staphylococcus aureus (7)<br>Streptococcus mitis (2)<br>Group B Streptococcus (1)<br>Streptococcus viridians (1)
Gram negative bacteria (N)	Pseudomonas aeruginosa (2)<br>Escherichia coli (1)<br>Bacteroides fragilis (1)<br>Citrobacter freundii (1)
Viruses (N)	Respiratory syncytial virus (2)<br>Influenza A (1)<br>Rhinovirus (1)<br>Human metapneumovirus (1)<br>Enterovirus (1)

Causative organisms isolated in patients. N gives the number of patients with a given proven infection.

Table 2. Baseline patient characteristics

Characteristic	PICU/sepsis (n=28)	PICU severe sepsis (n=17)	*P* value
Gender, n(%)			0.243
Female	12 (42.9)	7 (41.2)	
Male	16 (57.1)	10(58.8)	
Comorbidity present, n(%)	14 (50.0)	8 (47.1)	0.237
Age(yr)	9.5 (5.4)	12.0 (5.8)	0.156
Weight(kg)	33.3 (21.7)	42.0 (22.1)	0.212
PIM-2	3.0 (3.4)	6.9 (13.2)	0.145
WBC	16.4 (17.0)	15.5 (8.0)	0.847
Hgb	14.0 (14.3)	11.3 (1.6)	0.446
Hct	36.7 (19.4)	33.5 (4.9)	0.528
Plt	244.7 (135.5)	188.2 (104.4)	0.157
Na	164.0 (81.9)	137.8 (3.6)	0.206
K	8.0 (12.3)	3.6 (0.6)	0.161
Cl	126.0 (59.2)	108.9 (4.1)	0.250
HCO_3	32.0 (29.8)	19.5 (2.6)	0.100
BUN	15.4 (18.2)	14.8 (15.2)	0.911
Cr	4.0 (10.3)	0.9 (0.9)	0.224
Ang-1	2979.2 (4024.8)	2105.7 (3922.6)	0.490
Ang-2	4579.8 (3982.4)	15005.0 (19287.0)	0.010
Ang-2/Ang-1	7.9 (22.2)	58.1 (113.5)	0.032
VEGF	64.5 (60.3)	46.2 (49.5)	0.311

Statistical analysis of the baseline patient characteristics based on the evaluation distributions of the PICU/sepsis group and PICU severe sepsis group. Categorical variables, presented as count (percentage), were analyzed using Fisher exact test. Continuous variables, presented as mean (standard deviation), were analyzed using the two-tailed t test. *P* values are comparisons between two groups. Any significance level of *P* less than 0.05 is associated with the diagnosis.

Figure 1. Sample size by study day. Samples were obtained twice per day for the first 3 days and then once per day for the last 4 days, for a maximum of 7 days and 10 samples. Sample collection was discontinued when the patient was discharged from the PICU, after the 7-day study completion, or when the clinical team deemed it unnecessary to draw further labs for patient care.
